# Supplementary figures and images for: Application of visual communication in digital animation advertising design using convolutional neural networks and big data
Source: PeerJ Comput Sci. 2023 Jun 7;9:e1383. doi: 10.7717/peerj-cs.1383 (PMC10280566; doi:10.7717/peerj-cs.1383)

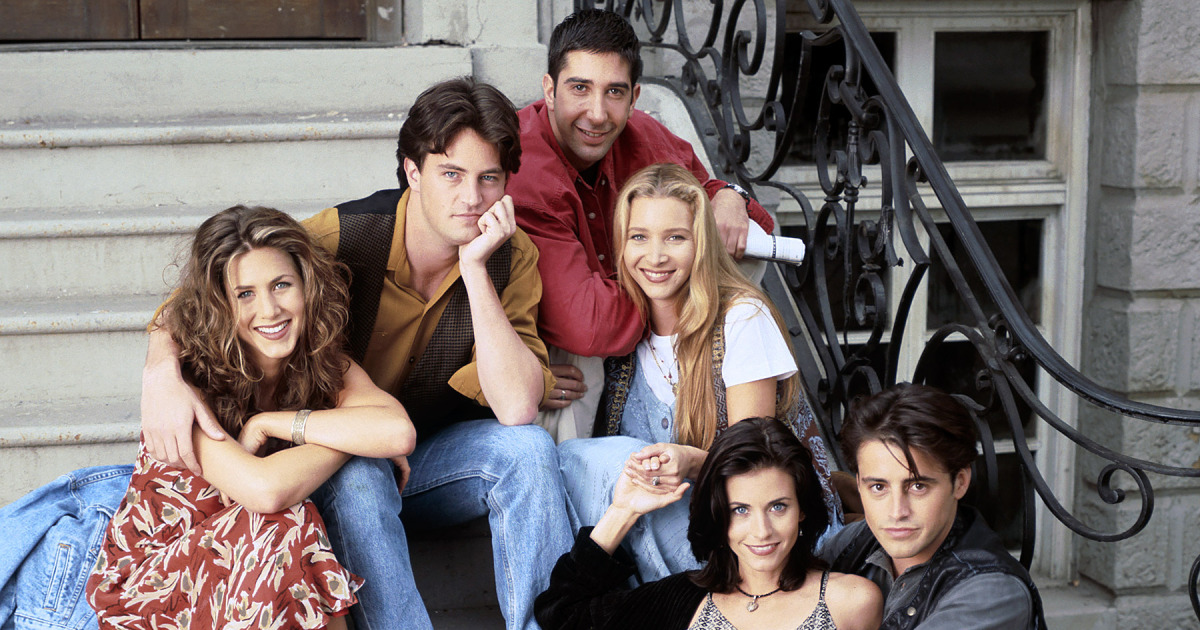

Supplement: Supplemental Information 1 [file peerj-cs-09-1383-s001.zip › Code/facenet_pytorch_local/data/multiface.jpg]

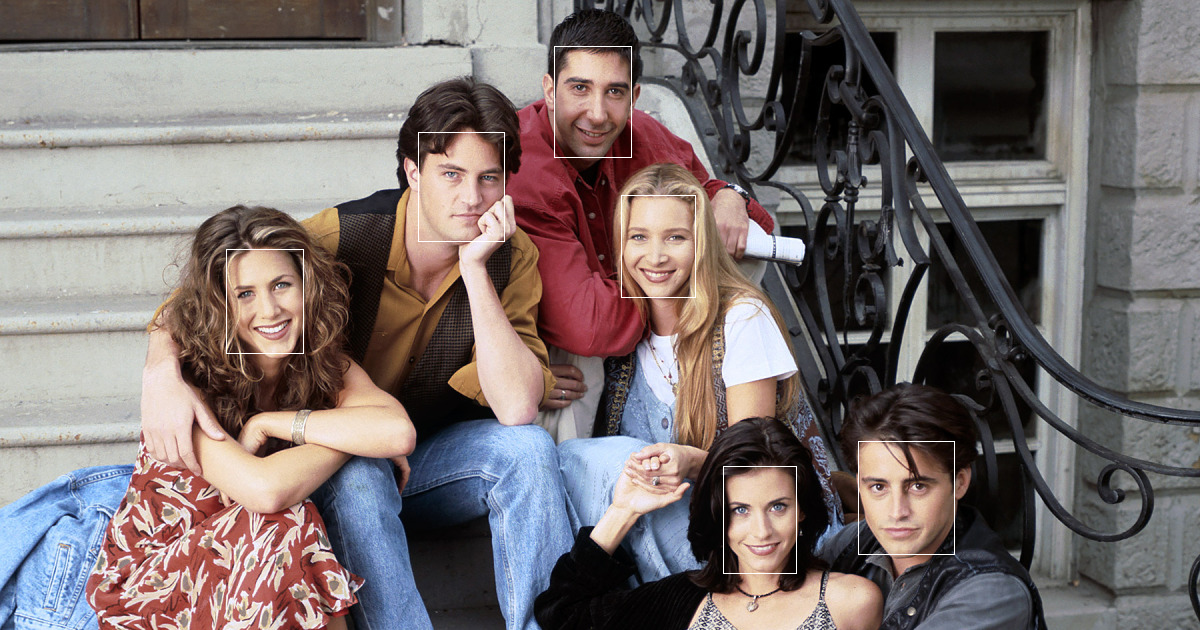

Supplement: Supplemental Information 1 [file peerj-cs-09-1383-s001.zip › Code/facenet_pytorch_local/data/multiface_detected.png]

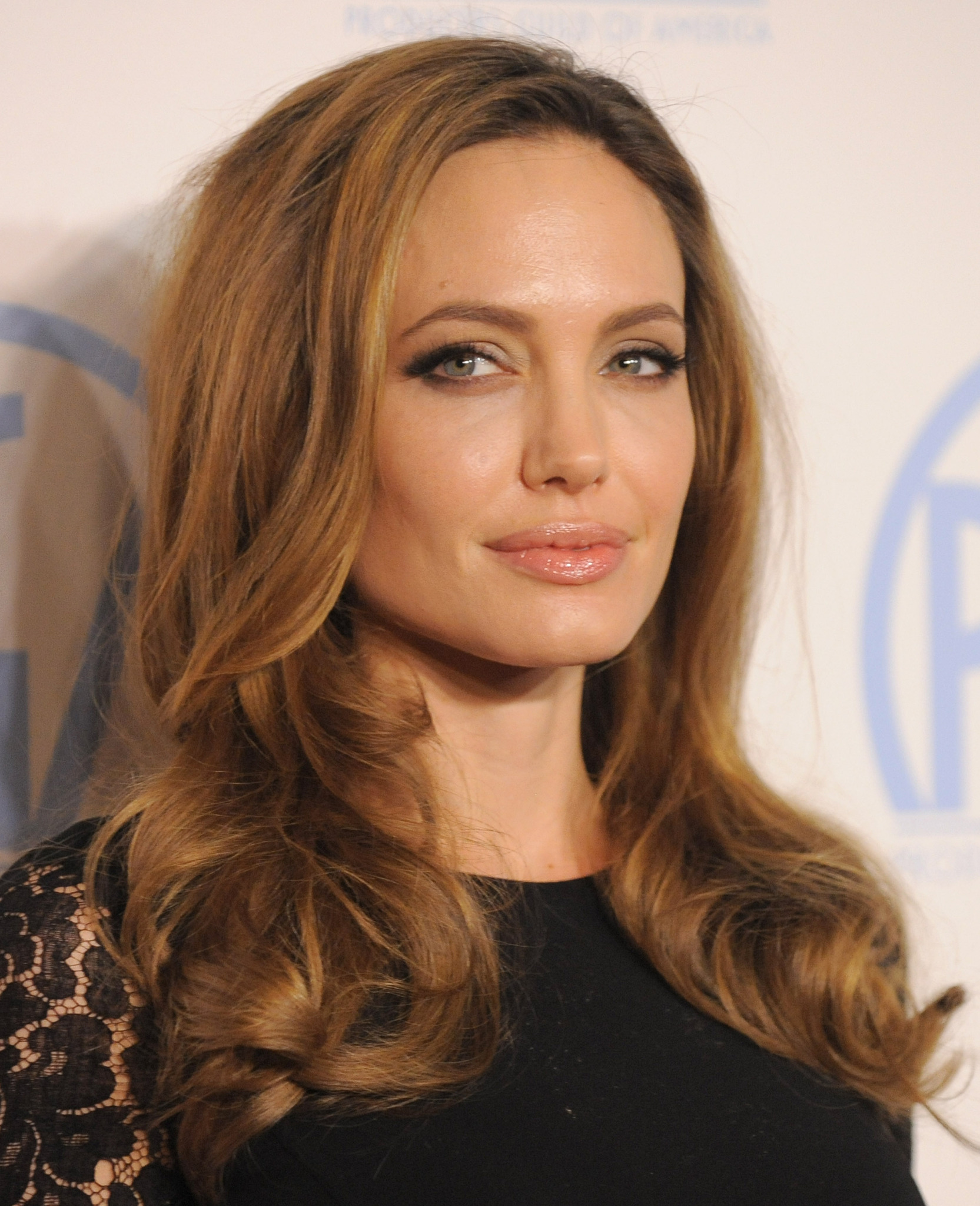

Supplement: Supplemental Information 1 [file peerj-cs-09-1383-s001.zip › Code/facenet_pytorch_local/data/test_images/angelina_jolie/1.jpg]

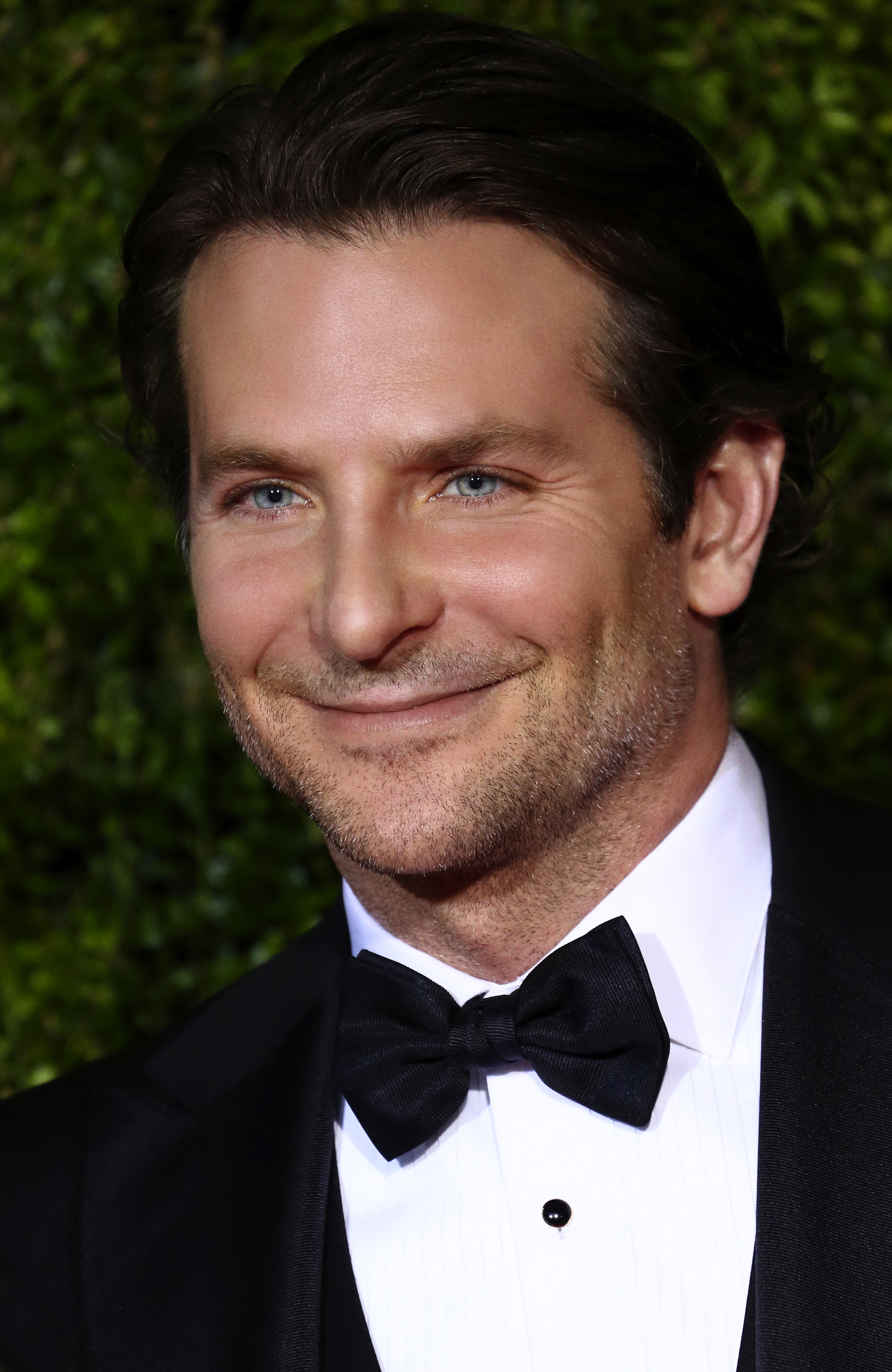

Supplement: Supplemental Information 1 [file peerj-cs-09-1383-s001.zip › Code/facenet_pytorch_local/data/test_images/bradley_cooper/1.jpg]

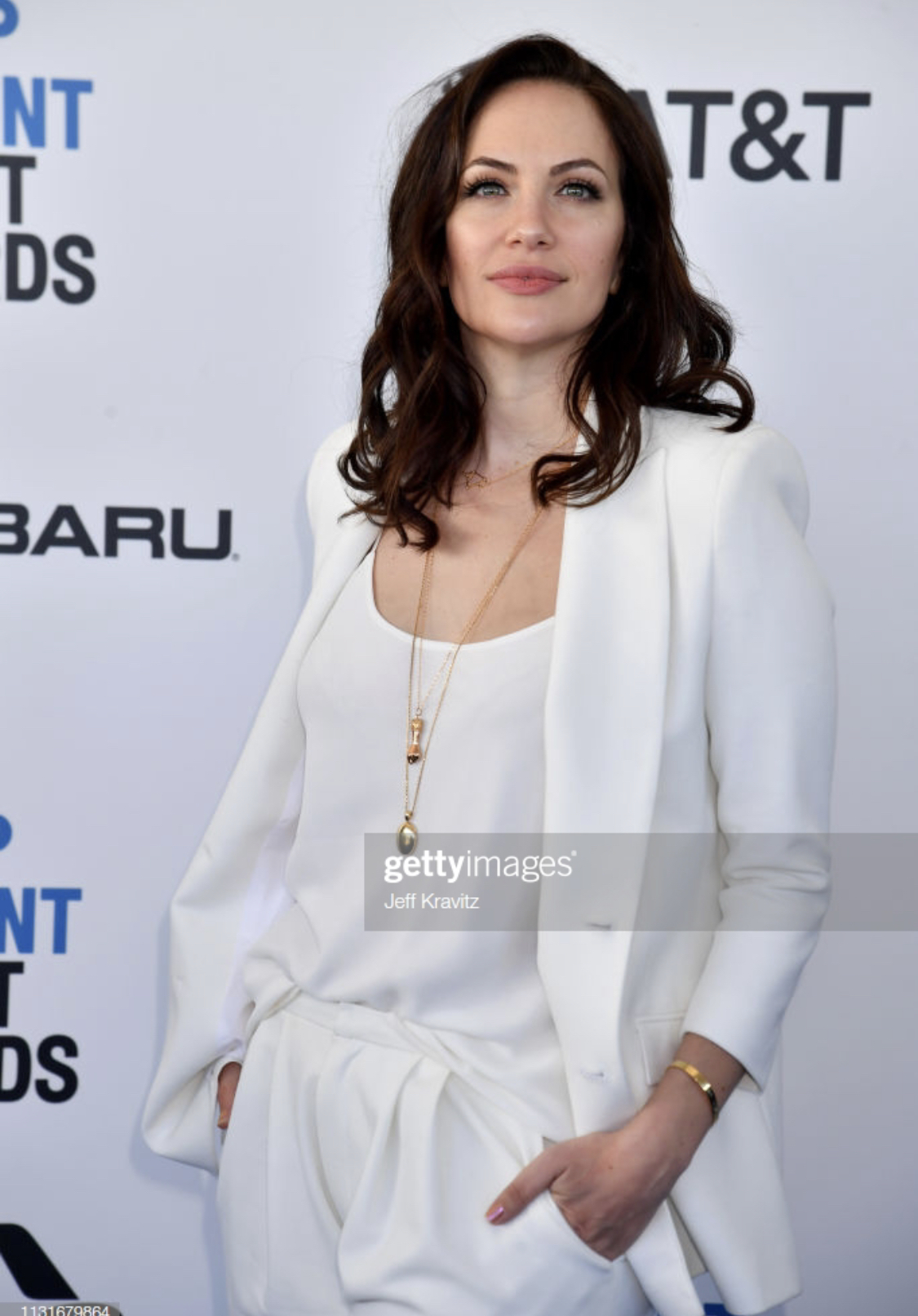

Supplement: Supplemental Information 1 [file peerj-cs-09-1383-s001.zip › Code/facenet_pytorch_local/data/test_images/kate_siegel/1.jpg]

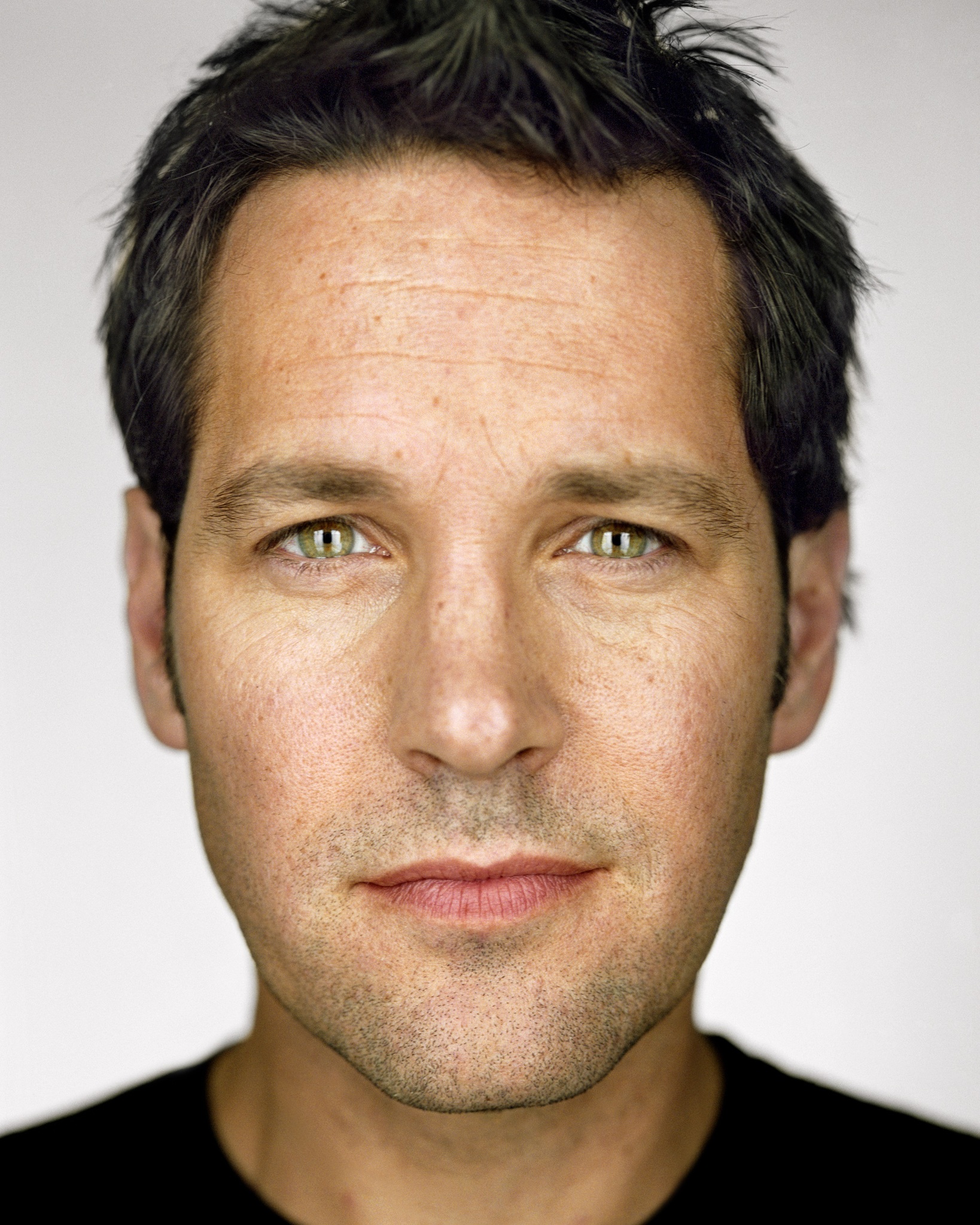

Supplement: Supplemental Information 1 [file peerj-cs-09-1383-s001.zip › Code/facenet_pytorch_local/data/test_images/paul_rudd/1.jpg]

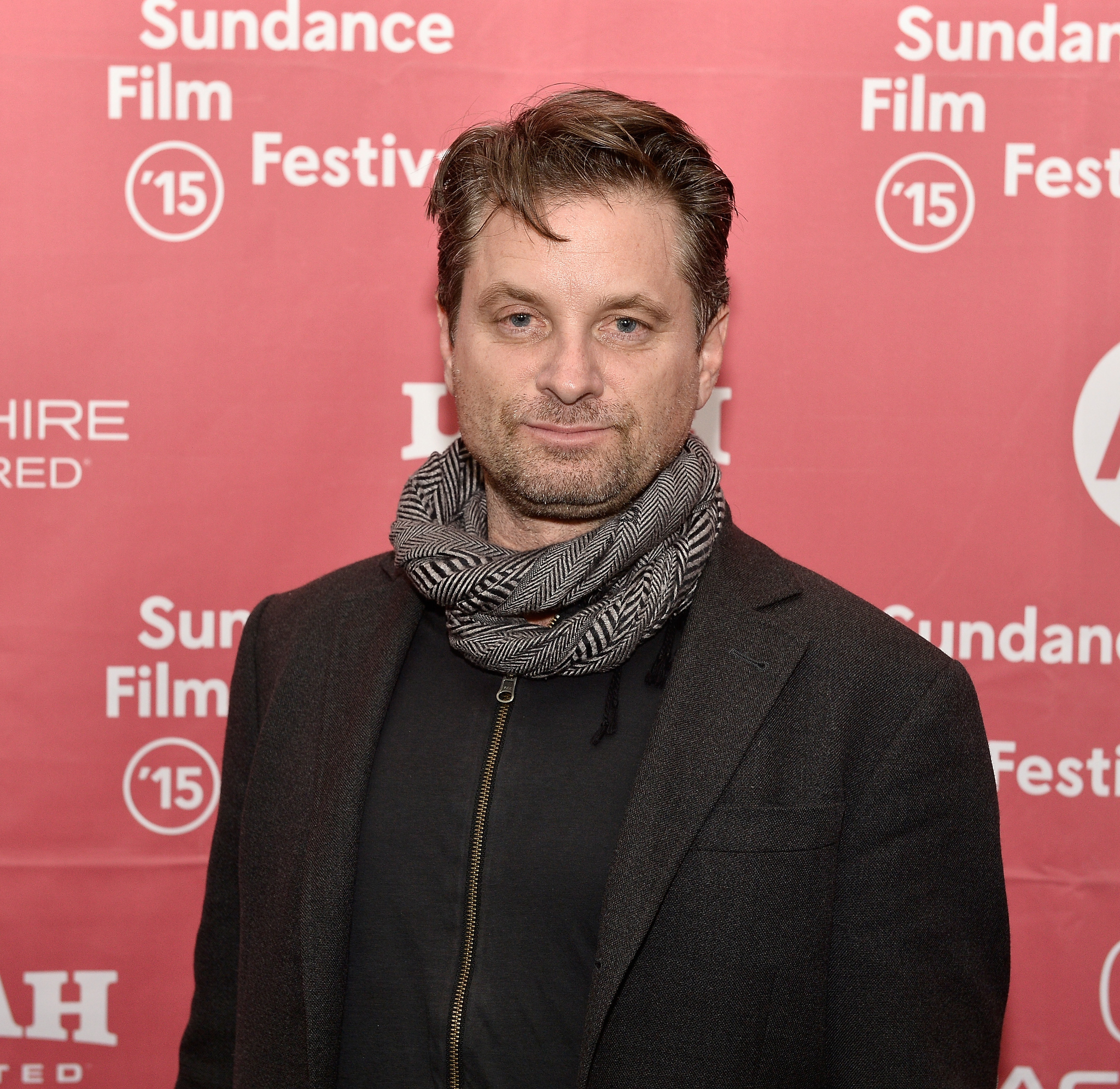

Supplement: Supplemental Information 1 [file peerj-cs-09-1383-s001.zip › Code/facenet_pytorch_local/data/test_images/shea_whigham/1.jpg]

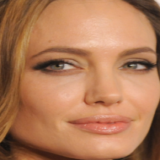

Supplement: Supplemental Information 1 [file peerj-cs-09-1383-s001.zip › Code/facenet_pytorch_local/data/test_images_aligned/angelina_jolie/1.png]

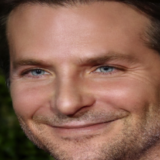

Supplement: Supplemental Information 1 [file peerj-cs-09-1383-s001.zip › Code/facenet_pytorch_local/data/test_images_aligned/bradley_cooper/1.png]

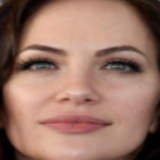

Supplement: Supplemental Information 1 [file peerj-cs-09-1383-s001.zip › Code/facenet_pytorch_local/data/test_images_aligned/kate_siegel/1.png]

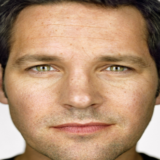

Supplement: Supplemental Information 1 [file peerj-cs-09-1383-s001.zip › Code/facenet_pytorch_local/data/test_images_aligned/paul_rudd/1.png]

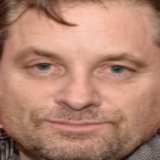

Supplement: Supplemental Information 1 [file peerj-cs-09-1383-s001.zip › Code/facenet_pytorch_local/data/test_images_aligned/shea_whigham/1.png]
